# Tree Circumference Changes and Species-Specific Growth Recovery After Extreme Dry Events in a Montane Rainforest in Southern Ecuador

**DOI:** 10.3389/fpls.2019.00342

**Published:** 2019-03-22

**Authors:** Volker Raffelsbauer, Susanne Spannl, Kelly Peña, Darwin Pucha-Cofrep, Kathy Steppe, Achim Bräuning

**Affiliations:** ^1^Institute of Geography, Friedrich Alexander University Erlangen-Nürnberg, Nuremberg, Germany; ^2^Department of Plant Physiology, University of Bayreuth, Bayreuth, Germany; ^3^Laboratorio de Dendrocronología y Anatomía de la Madera, Carrera de Ingeniería Forestal, Universidad Nacional de Loja, Loja, Ecuador; ^4^Laboratory of Plant Ecology, Department of Plants and Crops, Faculty of Bioscience Engineering, Ghent University, Ghent, Belgium

**Keywords:** stem diameter variations, dendrometer, tropical mountain rainforest, drought recovery, tree life form

## Abstract

Under drought conditions, even tropical rainforests might turn from carbon sinks to sources, and tree species composition might be altered by increased mortality. We monitored stem diameter variations of 40 tree individuals with stem diameters above 10 cm belonging to eleven different tree genera and three tree life forms with high-resolution dendrometers from July 2007 to November 2010 and additionally March 2015 to December 2017 in a tropical mountain rainforest in South Ecuador, a biodiversity hotspot with more than 300 different tree species belonging to different functional types. Although our study area receives around 2200 mm of annual rainfall, dry spells occur regularly during so-called “Veranillo del Niño” (VdN) periods in October-November. In climate change scenarios, droughts are expected with higher frequency and intensity as today. We selected dry intervals with a minimum of four consecutive days to examine how different tree species respond to drought stress, raising the question if some species are better adapted to a possible higher frequency and increasing duration of dry periods. We analyzed the averaged species-specific stem shrinkage rates and recovery times during and after dry periods. The two deciduous broadleaved species *Cedrela montana* and *Handroanthus chrysanthus* showed the biggest stem shrinkage of up to 2 mm after 10 consecutive dry days. A comparison of daily circumference changes over 600 consecutive days revealed different drought responses between the families concerning the percentage of days with stem shrinkage/increment, ranging from 27.5 to 72.5% for *Graffenrieda emarginata* to 45–55% for *Podocarpus oleifolius* under same climate conditions. Moreover, we found great difference of recovery times after longer-lasting (i.e., eight to ten days) VdN drought events between the two evergreen broadleaved species *Vismia cavanillesiana* and *Tapirira guianensis.* While *Vismia* replenished to pre-VdN stem circumference after only 5 days, *Tapirira* needed 52 days on average to restore its circumference. Hence, a higher frequency of droughts might increase inter-species competition and species-specific mortality and might finally alter the species composition of the ecosystem.

## Introduction

Global change, especially climate change, affects forests worldwide, with adverse effects on biodiversity and ecological services like carbon sequestration. Hence, understanding forest responses to climate variability is key to conservation and protection of forest ecosystems (Anderson-Teixeira et al., 2015). Especially, selective mortality of species can trigger long-term shifts in forest communities (Anderegg et al., 2013). Climate induced changes in forest composition are therefore an important scientific topic since several decades (Overpeck et al., 1990). Especially, drought as one of the most frequent climatic extremes on a global scale (Allen et al., 2009, 2015) and the accompanied increase in atmospheric vapor pressure deficit is, due to its partly devastating impact on forests, still under intense debate (Sass-Klaassen et al., 2016). According to climate modeling results (Fu et al., 2013), drought frequency and intensity in the tropics will increase in the near future. Recent studies on gross primary production and ecosystem respiration (Cavaleri et al., 2017) revealed that tropical forests may shift from carbon sinks to sources under drought conditions (Qie et al., 2017). Monitoring stem radial increment with high-resolution dendrometers can provide useful information if trees are in a state of active growth and carbon uptake, or if they are in a state of cambial inactivity due to drought stress (Steppe et al., 2015). However, there is still limited knowledge regarding the seasonal occurrence of positive or negative radial diameter or circumference variations of tree stems, especially at hourly or daily scale (De Swaef et al., 2015). Furthermore, the possibilities of the interpretation of dendrometer readings considering plant physiology are not fully exploited yet (Zweifel, 2016). These deficiencies are in particular valid for the tropics. Although a number of studies deals with tree growth in the tropics on a seasonal or annual level (e.g., Shimamoto et al., 2016; Wagner et al., 2016; Xu et al., 2016), only few studies analyze stem diameter variations of tropical trees on an intra-daily scale (e.g., Volland-Voigt et al., 2009; Butz et al., 2016; Spannl et al., 2016). To directly monitor the impact of drought on a forest and tree species specific response, it is necessary to have a close look at the individual tree level.

Disentangling the various factors leading to stem circumference changes, such as swelling and shrinkage of phloem, xylem and bark due to water potential and elastic properties of tissues (e.g., Rosner et al., 2009; Robert et al., 2014) and radial growth including cambial division and cell expansion are still a topic of ongoing research (e.g., Chan et al., 2015; Mencuccini et al., 2017) varying for each species. Modeling approaches offer one solution to solve this problem (Hölttä et al., 2010; Zweifel et al., 2014; De Swaef et al., 2015; Schippers et al., 2015; Steppe et al., 2015, 2016; Cocozza et al., 2018), however, complex models require numerous parameters with some of them, e.g., initial turgor phloem turgor pressure or elasticity of non-lignified xylem cells being challenging to acquire under field conditions. A different approach is defining growth empirically, if the diameter of a tree exceeds its previous day’s maximum (e.g., Deslauriers et al., 2003; Butz et al., 2016; van der Maaten et al., 2016; Zweifel et al., 2016). Although very promising, this approach has a limited capability considering extreme climatic events and considering drought coping strategies (e.g., succulence). Due to the constraints of the mentioned approaches we focus in the present study on analyzing daily stem diameter responses to drought between different tropical tree species and families of different plant functional types regarding their resilience and ability to cope with dry spells of varying length.

Analyzing the impact of climatic extremes on tree circumference provides important insights into tree functioning and carbon budget. Hence, analyzing responses of stem girth to extreme meteorological events is an important variable in climate-growth-modeling and helps to improve existing models (Siegmund et al., 2016). Stem circumference changes are thus a valuable indicator for analyzing drought legacy effects and carbon cycle balance (Anderegg et al., 2015) and tree mortality risks which rapidly increase under extreme drought conditions (Meir et al., 2015).

In this paper we analyze one of the largest dendrometer datasets existing in the tropics. Our study is separated into three parts. After examining the general response of stem size variations to climate parameters, we first analyzed how the different tree species in a moist tropical mountain forest in South Ecuador respond to different durations of rare dry spells. Second, we analyzed species-specific differences in growth dynamics and drought response over a 600-day period. Third, we compared recovery from drought in two contrasting tree species *Tapirira* and *Vismia* and draw inferences about possible consequences for forest composition in view of expected climate change. All add up to the main research question, how different humid mountain rainforest tree species respond to drought stress and if some species are better adapted to a possible higher frequency and increasing duration of dry periods.

## Materials and Methods

### Study Site and Local Climate

The study was conducted in a lower montane rainforest (Homeier, 2004) on the eastern declivity of the Cordillera Real in southern Ecuador. The study site is located at the northern slope of the Podocarpus National Park (3°58′S, 79°04′W) on an elevation of ca. 2000 m a.s.l. The soils at the study site are very heterogeneous, with humic cambisols dominating on the slopes (Wilcke et al., 2008). The studied forest is located in a biodiversity hotspot and hosts more than 280 tree species, with special importance of the families *Melastomataceae*, *Lauraceae*, *Rubiaceae*, and *Euphorbiaceae* (Homeier et al., 2004).

We used climate datasets used from several local meteorological stations with a maximal distance to our study site of approximately 500 m. Mean annual air temperature is 15.5°C (Fries et al., 2009), and average annual rainfall is 2200 mm. Over the year, the region experiences three different seasons characterized by changing wind directions and precipitation amounts: between January and April, humid air masses from SE and NE directions dominate, with an average rainfall of around 800 mm. During May to August, northeasterly winds dominate, bringing about 1000 mm precipitation. Finally, during September to December, only 400 mm of rainfall are recorded. In this phase the climate phenomenon “Veranillo del Nino” (VdN) may occur, which is caused by a pronounced low pressure system east of the Andes weakening the dominating trade winds over our study area. This leads to reduced cloud cover and higher amounts of solar irradiance at our study site (Emck, 2007; Bendix et al., 2008, Rollenbeck et al., 2011). Due to increased outgoing longwave radiation (OLR) under clear sky during the night, the daily temperature range increases to almost 25K, spanning from 2.4°C during nighttime to 27.1°C during daytime.

Longer dry intervals (7–9 days) almost exclusively occur during VdN events, except one drought event during 30.06.2010–06.07.2010 (Supplementary Table S1). In oral lore of local farmers, VdN reoccurs every year in the first week of November, but its timespan has become more irregular in recent years. The preconditions for its occurrence, i.e., a change in the dominant easterly wind direction are also fulfilled in October, December and January (Emck, 2007), and four of the other registered dry spells arose in those months. Other dry spells with four or more days without precipitation occurred in July and August (Supplementary Table S1).

### Dendrometer Measurements and Tree Species

The data for this study were collected during two different study periods. From July 2007 to November 2010, tree growth dynamics and radial stem changes were measured every 30 min with electronic point dendrometers (Type DR, Ecomatic, Germany). From March 2015 to December 2017, logging band dendrometers (LBDs) with a built-in thermometer (DRL26, EMS Brno) were used. For comparability of the two dendrometer types, we converted the data of the point dendrometers into circumference values using the formula: circumference = 2 × π × radius, assuming symmetrical stem geometries of the studied individuals. Due to latest calibration tests, the used dendrometer types show different thermal sensor coefficients of 3.29092 μm°C^-1^ for the type DR and 0.0054 μm °C^-1^ for the type DRL26, respectively (von der Crone et al., pers. comm., work under review). Since the maximum temperature differences between consecutive nights in our study area do not exceed 3°C, no temperature correction was needed for the values derived from band dendrometers. Due to the higher thermal sensor coefficient of 3.29 μm°C^-1^ of the point dendrometers, their maximum error is the range of up to 62 μm in circumference. An error in this range does not question our main results. Data recorded by dendrometers of DR-type have only been used in the first part of the study. In all cases, dendrometers were installed at breast height (approx. 1.30 m). In case of thick-barked species, parts of the outer bark were removed without injuring the cambial zone to minimize the influence of bark swelling and shrinking due to water uptake or loss. Since active growth by wood formation and stem swelling due to water uptake cannot be differentiated by dendrometer data we will use the term “increment” in the following for any kind of stem diameter increase. To investigate the impact of drought on different species we selected dry intervals with a minimum of four consecutive days without rainfall during the periods July 2007 to November 2010 and March 2015 to December 2017. We analyzed the averaged stem shrinkage rates during periods from 4 to 9 days. To calculate stem shrinkage, only the daily maximum stem diameter values (Deslauriers et al., 2007) were compared. In order to create a more robust dataset, the values regarding the dry spells include the data of all droughts with the minimum amount of days without precipitation.

In total, 40 tree individuals with stem diameters above 10 cm belonging to eleven different tree genera and three tree life forms were studied (Table 1). Stem diameter variations of trees of the same genus that belong to the same plant functional type were averaged to increase replication, this applies for the genera of the Lauraceae family, namely *Persea*, *Nectandra*, and *Ocotea*. Hereafter, we use the genus names for the groups of trees comprising more than one species and the species names, if the genus is represented by only one species. For *Prumnopitys*, our data regarding dry spells is limited to 6 days due to data logger problems.

**Table 1:** Characteristics of studied tree species equipped with electronic dendrometers.

Tree family	Tree species	No. of individuals	Life form
Bignoniaceae	*Handroanthus chrysanthus*	4	deciduous broadleaved
Meliaceae	*Cedrela montana*	5	deciduous broadleaved
Fabaceae	*Inga acreana*	3	evergreen broadleaved
Lauraceae	*Persea spp.*	4	evergreen broadleaved
	*Nectandra spp.*	4	evergreen broadleaved
	*Ocotea spp.*	2	evergreen broadleaved
Anacardiaceae	*Tapirira guianensis*	4	evergreen broadleaved
Hypericaceae	*Vismia cavanillesiana*	5	evergreen broadleaved
Melastomataceae	*Graffenrieda emarginata*	3	evergreen broadleaved
Podocarpaceae	*Podocarpus oleifolius*	4	evergreen coniferous
	*Prumnopitys montana*	2	evergreen coniferous


### Statistical Analysis

The dendrometer data were scanned for outliers and measurement artifacts (exceeding 0.5 mm stem circumference change within 30 min). For stem shrinkage and increment calculation we took the maximum daily circumference/diameter values and subtracted the values from the subsequent day. The distinguished groups for the second part of the study (Figure 2) differed significantly from each other (tested by Welch ANOVA test). We standardized the data with the formula z = (x-μ) /σ, where x is the measurement value, μ is the arithmetic mean and σ is the standard deviation. For better visual comparison, we divided the outcome by 20. The relationships between changes in stem circumference and climatic variables were tested with linear correlations. All statistical analyses were conducted with the R programming language (R Development Core Team, 2015) thought the software RStudio version 3.2.2.

## Results

### Tree Species Response to Dry Spells of Different Length

**FIGURE 1:**
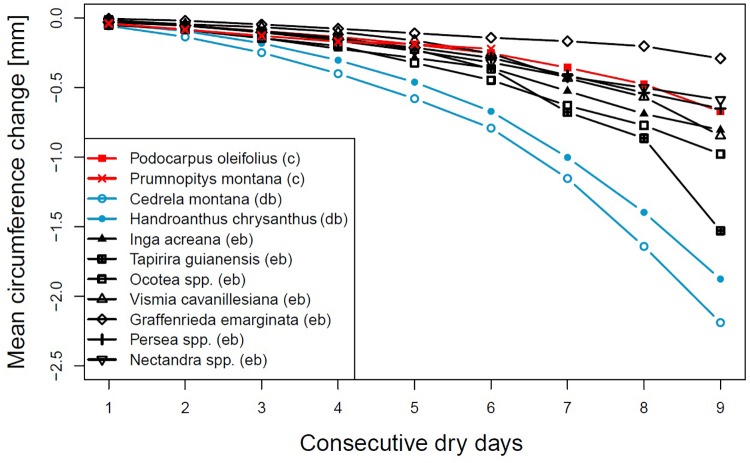
Mean circumference changes after consecutive rainless days for different genera or species. Evergreen broadleaved (eb, black), coniferous (c, red) or deciduous broadleaved (db, lightblue).

Evergreen broadleaved and coniferous species showed a lower rate of stem diameter shrinkage in relation to dry interval length than the deciduous broadleaved species *H. chrysanthus* and *C. montana*, with average circumference losses of 1.876 and 2.190 mm after nine dry days, respectively (Figure 1). *G. emarginata* lost least of their circumference, with a maximum loss of only 0.290 mm after nine consecutive dry days. *T. guianensis* first showed a circumference loss similar to most of the evergreen broadleaved species, but after the fifth rainless day shrinkage rates strongly increased and summed up to 1.528 mm after the ninth rainless day. Also noticeable is the strong circumference loss of *V. cavanillesiana* from the eighth to the ninth day without rainfall. The two coniferous species showed no differences compared to most of the evergreen broadleaved species.

**FIGURE 2:**
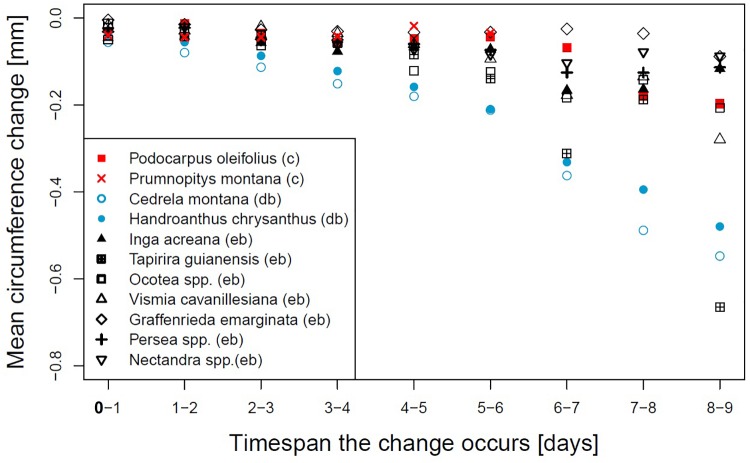
Daily mean circumference change on subsequent days for different genera or species. Evergreen broadleaved (eb, black), coniferous (c, red) or deciduous broadleaved (db, lightblue).

A closer look to the day-to-day circumference changes (Figure 2) reveals that for the two deciduous broadleaved species the circumference loss was constantly increasing with increasing drought duration. *T. guianensis* showed lower losses from the fifth to sixth and seventh to eighth day compared to the day before and shrunk 0.662 mm on the last day, which was the strongest observed shrinkage of all species. *V.* *cavanillesiana* showed a strong loss of circumference of 0.279 mm on the ninth day, which was more than double the amount of any day before. The shrinkage of *Inga acreana* declined after the seventh day, and also *Nectandra* and *Persea* showed maximum shrinkage from the sixth to the seventh rainless day.

**FIGURE 3:**
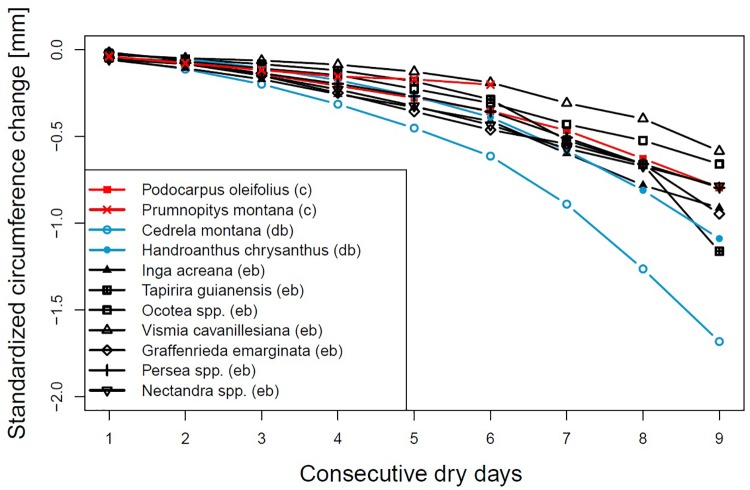
Standardized circumference changes after consecutive rainless days for different genera or species. Evergreen broadleaved (eb, black), coniferous (c, red) or deciduous broadleaved (db, lightblue).

To make the values more comparable, we used z-transformation (Figure 3). *Cedrela montana* also showed the highest standardized change but *Handroanthus chrysanthus* moved closer to the other species. *Graffenrieda emarginata* which showed the lowest shrinkage in absolute values now blended in with the other data.

### Species-Specific Response of Stem Size Variations to Climate Variables, Differences of Growth Dynamics and Drought Response

For the comparison of the overall growth behavior of the studied species, we selected a timespan from 12.04.2016 to 01.12.2017 (599 days) in which no missing dendrometer measurement values, which might compromise the results, occurred. The species *Handroanthus chrysanthus*, *Cedrela montana*, *Inga acreana, Prumnopitys montana* and the genera *Persea and Nectandra* had to be excluded from this comparison because they were not instrumented during the second measurement period from March 2015 to December 2017.

In Table 2, correlations between daily stem circumference changes and different climate parameters are shown. Mean relative humidity and vapor pressure deficit showed the highest correlation values. Temperature parameters and global radiation were also significantly correlated. Precipitation of the current day showed higher correlations with stem size variations than precipitation of the previous day. Soil moisture was measured at a site close to our study trees (ca. one kilometer distance, same elevation). Over the year, soil water content measured 10 cm below ground only varied between 35.7 and 48.7% over the year (Moser et al., 2008). Factors dampening soil water content fluctuations are the high amount of annual precipitation, the regular rainfall distribution (apart from the short dry spells), the high clay content of the deeply developed soils, and the dense rainforest canopy preventing solar insulation of the soil surface. Despite these factors, soil water content and soil moisture tension are crucial variables for trees and influence water dependent systems e.g., by buffering drought phases or prolonging the experienced drought past the first rainfalls until the soil are replenished. Because soil moisture data are not available for our study area over the studied time periods, we are not able to quantify this possible error.

**Table 2:** Correlations between daily circumference changes of different tree species with climate parameters.

	rHmean	P	P-1	T Mean	T min	T. max	VPD	gRad
*Ocotea*	0.72^∗∗∗^	0.50^∗∗∗^	0.22^∗∗^	–0.45^∗∗∗^	0.41^∗∗∗^	–0.40^∗∗∗^	–0.72^∗∗∗^	–0.52^∗∗∗^
*Graffenrieda*	0.70^∗∗∗^	0.52^∗∗∗^	0.23^∗∗^	–0.45^∗∗∗^	0.40^∗∗∗^	–0.41^∗∗∗^	–0.70^∗∗∗^	–0.50^∗∗∗^
*Tapirira*	0.71^∗∗∗^	0.51^∗∗∗^	0.19^∗^	–0.36^∗∗∗^	0.49^∗∗∗^	–0.40^∗∗∗^	–0.69^∗∗∗^	–0.51^∗∗∗^
*Vismia*	0.61^∗∗∗^	0.53^∗∗∗^	0.18^∗^	–0.31^∗∗∗^	0.38^∗∗∗^	–0.29^∗∗∗^	–0.59^∗∗∗^	–0.41^∗∗∗^
*Podocarpus*	0.67^∗∗∗^	0.57^∗∗∗^	0.14	–0.40^∗∗∗^	0.40^∗∗∗^	–0.40^∗∗∗^	–0.66^∗∗∗^	–0.51^∗∗∗^


*rHmean, daily mean relative humidity; *P*, daily precipitation; *P-1*, previous day precipitation; *T Mean*, daily mean temperature; *T min*, daily minimum temperature; *T max*, daily maximum temperature; *VPD*, vapor pressure deficit; *gRad*, global radiation. ^∗^95% significance; ^∗∗^99% significance; ^∗∗∗^99.9% significance.*

In spite of the identical climatic conditions, differences in the percentages of days with increment or shrinkage are apparent. *G. emarginata* showed the highest percentage of days with stem increment (68.1%) and also the lowest percentage of days with shrinkage (28.5%). These values are followed by *V.* *cavanillesiana* (65/29.8%), *T. guianensis* (62.3/35.8%), *Ocotea* (58.3/38.7%) and *P. oleifolius* (55.5/40.3%). The percentages missing to 100% is stagnation of circumference (Supplementary Figure S1).

For the comparison of the maximum daily increment and maximum daily shrinkage we averaged the three highest values to lower the influence of outliers. Table 3 shows a distinction into three groups: *Ocotea* with equal maximum values of shrinkage and increment, *G.* *emarginata* and *T. guianensis* with the maximum daily shrinkage exceeding the maximum daily increment, and *V.* *cavanillesiana* and *P. oleifolius* with dominant maximum daily increment values. In contrast to the maximum values, *Ocotea* and *G.* *emarginata* are the only genera which showed higher values of mean daily shrinkage than mean daily increment (Table 3).

**Table 3:** Species specific differences in growth dynamics.

	I_max	SD±	S_max	SD±	I_me	SD ±	S_me	SD ±	I_sum	SD ±	S_sum	SD ±
Graffenrieda	0.078	0.006	0.191	0.092	0.016	0.000	0.023	0.012	6.110	1.745	2.285	0.489
Ocotea	0.294	0.056	0.294	0.022	0.036	0.000	0.039	0.011	14.349	3.819	7.907	0.260
Podocarpus	0.243	0.054	0.160	0.027	0.026	0.003	0.023	0.004	8.718	0.863	5.691	1.301
Tapirira	0.338	0.048	0.357	0.076	0.046	0.007	0.040	0.005	17.260	2.150	8.733	1.822
Vismia	0.300	0.131	0.235	0.037	0.029	0.011	0.022	0.007	11.603	0.828	3.839	0.678


*I_max, Average daily maximum increment; S_max, Average daily maximum shrinkage; I_me, Average daily mean increment; S_me, Average daily mean shrinkage; I_sum, Average cumulated increment; S_sum, Average cumulated shrinkage; SD, standard deviation.*

When considering cumulated changes, the species showing the highest increment also showed the highest shrinking rates (Table 3). The mean net circumference gain was 6.44 mm for *Ocotea*, 3.82 mm for *G. emarginata*, 3.03 mm for *P. oleifolius*, 8.53 mm for *T. guianensis*, and 7.76 mm for *V. cavanillesiana*, respectively. For better visualization of the species differences, the figures respective to Table 3 can be found in the Supplementary Figures S2–S4.

### Species-Specific Recovery From Drought

When comparing species and families during the 60 days without precipitation over the study period of 539 days, all groups showed very similar percentages of days with increment, ranging from 15.8 to 18.4%. Due to this homogenous behavior during dry days, we assumed that an important difference in the response to climate may exist following the dry spells. We therefore analyzed in detail the recovery times of the different species. Two species are standing out because of their contrasting response during and after drought, namely *T. guianensis* and *V. cavanillesiana*.

**FIGURE 4:**
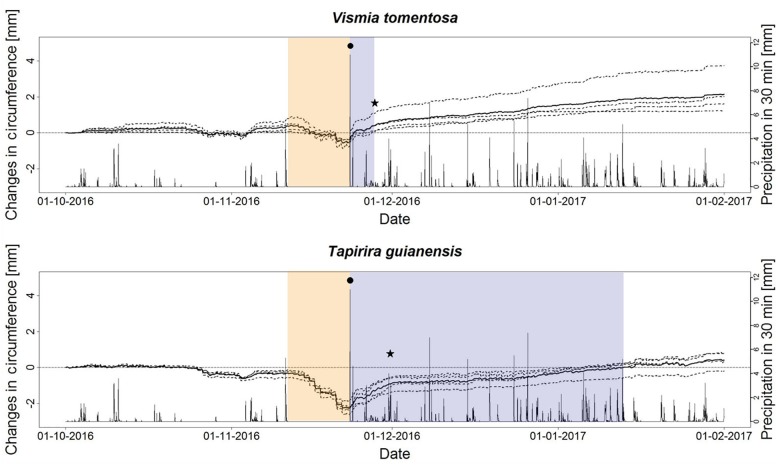
Circumference change from October 2016 to February 2017 of *Vismia cavanillesiana* and *Tapirira guianensis*. Dotted lines show individual trees; solid lines show the corresponding mean values; orange backgrounds indicate the dry spell; blue backgrounds mark the time to reach the pre-drought maximum; (•) first rainfall after dry spell; (★) switching from replenishment to growth.

Both species showed a very consistent response pattern among different individuals (Figure 4). Their responses considering the VdN drought lasting from 13 to 23 November 2016 were at first very similar, starting with a steady circumference loss which was more pronounced in *T. guianensis.* However, very different responses occured concerning the replenishing phase after the first rainfall on the 23 November 2016. While the circumference of all *V. cavanillesiana* individuals rose immediately, the individuals of *T. guianensis* showed a much slower pattern of stem replenishment. The most distinct differenence beween the two species was the time needed to reach the stem diameter preceding the VdN phase. On average, *V. cavanillesiana* needed only 4 days to regain its pre-drought circumference. In contrast, *T. guianensis* regained only 50% of its losses within 4 days, and it took until early- January (45 days) until the pre-drought stem diameter was regained, but already at the beginning of December the species showed a flattening of the stem increment curve (★).

## Discussion

The rates of stem circumference shrinkage in relation to the dry time interval, the net circumference change between subsequent days and the standardized shrinkages (Figure 1–3) highlighted that evergreen broadleaved and coniferous species are able to regulate stem water loss after a couple of sunny and dry days, probably by the closure of leaf stomata (Ying et al., 2015). If the duration of a dry spell exceeds four consecutive days, differences between families became apparent. *G.* *emarginata* appeared very well adapted, showing the smallest absolute stem diameter shrinkage but standardized, it turned more to average. *P. oleifolius* showed the second smallest stem diameter change in response to ongoing drought, very similar to *Ocotea* and *Inga*. *V.* *cavanillesiana* responded very alike, but the daily changes of the last three dry days indicated differences in response, probably due to change in drought-coping adaptations like different degrees of leaf-water potential regulation (Ryan, 2011). The decreasing circumference loss between the seventh to the eighth dry day and the much higher loss on the ninth day could be an indication that a threshold of water loss was reached. The same assumption applies to *T. guianensis*, showing the maximum change on a single day of all investigated species.

The deciduous broadleaved species *H. chrysanthus* and *C. montana* share the ability of leaf shedding to regulate the risk of water loss by enhanced transpiration. The latter species showed during the timespans in which most of the dry spells occur around 50% foliage (Bendix et al., 2006), but both species showed the highest values of circumference loss from the very beginning of a dry spell. Even after 9 days without precipitation, stem circumferences constantly decreased with still increasing rates. This led us to speculate that if dry intervals occur during the growing period, when both deciduous species are fully foliated, these species may suffer even more from drought. As visualized by the standardized values *C. montana* is more sensitive and vulnerable to droughts compared to *H. chrysanthus*.

The significant correlations of precipitation, relative humidity and vapor pressure deficit with our stem circumference measurements are consistent with previous studies (e.g., Deslauriers et al., 2007; Bräuning et al., 2009; Volland-Voigt et al., 2009; Butt et al., 2014). The majority of the climatic parameters are highly inter-correlated, like e.g., global radiation and temperature.

Consistent with the small absolute response during the dry spells, *G.* *emarginata* showed the highest percentage of days with stem increment. However, when shrinkage occurred, the maximum and the mean shrinkage values exceeded the respective increment value. This seems not problematic due to the high percentage of days with net increment, and hence we do not classify the species as vulnerable. *G.* *emarginata* is also the second slowest growing species with the lowest cumulated increment but also with the lowest cumulated shrinkage. Slow growth may be related to drought tolerance as resource-conservative strategy (Ouédraogo et al., 2013).

*P. oleifolius* showed the lowest percentage of days with increment, but no peculiarities were found in its responses during drought. The increment maxima and mean values were both higher than their shrinkage values. The mean net growth during the analyzed timespan was the lowest with 3.03 mm. Hence, *Podocarpus* is demanding concerning climate conditions, but this is compensated by higher mean and maximum increment values during suitable climate conditions.

*Ocotea* is also unobtrusive regarding its dry spell responses and show an average percentage of days with increment compared to the other species. Maximum shrinkage values are equal to the increment maxima. The daily mean increment is lower than the mean shrinkage. This could be, in contrast to *G. emarginata*, potentially problematic for the family because of the lower percentage of days with increment.

*V.* *cavanillesiana* showed some distinctive features during drought, i.e., the percentage of days with shrinkage is only 29.8%. The values of mean and maximum increment are higher than the respective shrinkage. With 7.76 mm, the species’ net growth is the second highest of the analyzed species. *T. guianensis* shows the same features as *V.* *cavanillesiana*, but more intense. Overall, *T. guianensis* showed the highest maximum shrinkage values of all investigated species, but also net growth is highest, with 8.53 mm.

The flattening of the growth curve for *T. guianensis* indicates a switch from stem water replenishment into a usual growth pattern (Figure 4). The lower absolute stem circumference may be explained by embolism of xylem cells which reduces on the one hand the ability of expansion of the stem by replenishment of xylem cells with water and hence build-up of turgor, and on the other hand causes hydraulic dysfunction lowering the conductance of the stem. Recovery from a drought can be interfered by xylem cavitation (Luo et al., 2016). Other drought stress effects that may negatively affect post-drought growth despite favourable growth conditions include damaged organelle structures, decreased photosynthetic activity or induced chlorophyll degradation (Ying et al., 2015). *T. guianensis* seems therefore to be vulnerable to longer or higher frequency droughts. In our study, we determined similar responses between *T. guianensis* individuals but it is important to take into consideration that embolism resistance may vary intraspecifically (Anderegg, 2015). To sum up our findings and to answer the main research question, we can state that there is a difference in drought response between deciduous and evergreen trees regarding the absolute amount of circumference loss. However, when considering standardized values of stem shrinkage, the deciduous *C. montana* shows high drought sensitivity and vulnerability. Also *Tapirira* becomes more vulnerable with increasing duration of dry spells. Regarding the species’ responses during the 600 days drought and non-drought comparison, we consider *Ocotea* and again *Tapirira* as most vulnerable to drought. Our last analysis verified *Tapirira* being highly vulnerable to longer lasting droughts, resulting in a non-replenishable loss of stem circumference.

To make inferences about possible effects of the detected differences in drought response about possible species composition changes of the entire ecosystem or about species-specific mortality risk, a key research need is determination of species-specific thresholds of xylem embolism which are recoverable or not (Hartmann et al., 2018). Understanding the mechanisms of drought response, survival and mortality will be critical for predicting tree response to a changing climate (Ryan, 2011). This needs to be taken into consideration also regarding the carbon sink or source issue because special patterns of moist tropical forest carbon storage are primarily driven by mortality (McDowell et al., 2018).

Of interest for future research is if nonlethal droughts can stimulate drought resilience, since plants typically respond by acclimation of key hydraulic parameters like leaf area (reduction), root and sapwood area (increase), and cavitation resistance (increase), which should protect trees against future droughts (McDowell et al., 2008). Considering that the intact forest sink is declining in size, and that tropical forests may switch to become a net carbon source (Mitchard, 2018), more research in different tropical forest ecosystems is of utmost importance.

## Data Availability

The datasets generated for this study are available on request to the corresponding author.

## Author Contributions

AB and VR developed the concept of the manuscript. VR and KP collected the data in the field. VR and SS contributed statistics and graphics. All authors contributed to the text writing.

## Conflict of Interest Statement

The authors declare that the research was conducted in the absence of any commercial or financial relationships that could be construed as a potential conflict of interest.

## Supplementary Material

TABLE S1 Number of dry intervals and the dates they occurred.

FIGURE S1 Percentage of different circumference changes (%) for different genera.

FIGURE S2 Daily increment and shrinkage maxima (mm) for different genera with standard deviations.

FIGURE S3 Daily increment and shrinkage means (mm) for different genera with standard deviations.

FIGURE S4 Daily increment and shrinkage sums (mm) for different genera with standard deviations.
