# Supplementary material for: Tree Circumference Changes and Species-Specific Growth Recovery After Extreme Dry Events in a Montane Rainforest in Southern Ecuador
Source: Front Plant Sci. 2019 Mar 22;10:342. doi: 10.3389/fpls.2019.00342 (PMC6439692; doi:10.3389/fpls.2019.00342)
Supplement: TABLE S1 — Number of dry intervals and the dates they occurred. [file Table_1.docx]

Table S1. Number of dry intervals and the dates they occurred.

| Length of dry interval (days) | no. of dry intervals of respective length during 2007-2010 | no. of dry intervals of respective length during 2015-2017 | Values used for the study  2007-2010 | Values used for the study  2015-2017 | Time spans when dry spells occurred |
| --- | --- | --- | --- | --- | --- |
| 4 | 3 | 5 | 11 | 14 | 21.-24.07.07;  21.-24.08.07;  08.-11.09.10  13.-16.01.16;  17.-20.01.17;  11.-14.02.17;  16.-19.02.17;  21.-24.06.17; |
| 5 | 2 | 4 | 8 | 9 | 16.-20.12.08;  06.-10.08.10;  08.-12.01.16;  30.10.-03.11.16;  11.-15.07.17;  01.-05.11.17 |
| 6 | 1 | 3 | 6 | 5 | 19.-24.11.10;  30.06-05.07.16;  23.-28.10.16;  14.-19.11.17; |
| 7 | 3 | 0 | 5 | 2 | 13.-19.11.08;  30.06.-06.07.10;  03.-09.11.10 |
| 8 | 0 | 1 | 2 | 2 | 31.10 - 07.11.15 |
| 9 | 2 | 1 | 2 | 1 | 22.10-30.10.10;  31.10-08.11.10  13. - 22.11.16 |
