# Supplementary figures and images for: Tree Circumference Changes and Species-Specific Growth Recovery After Extreme Dry Events in a Montane Rainforest in Southern Ecuador
Source: Front Plant Sci. 2019 Mar 22;10:342. doi: 10.3389/fpls.2019.00342 (PMC6439692; doi:10.3389/fpls.2019.00342)

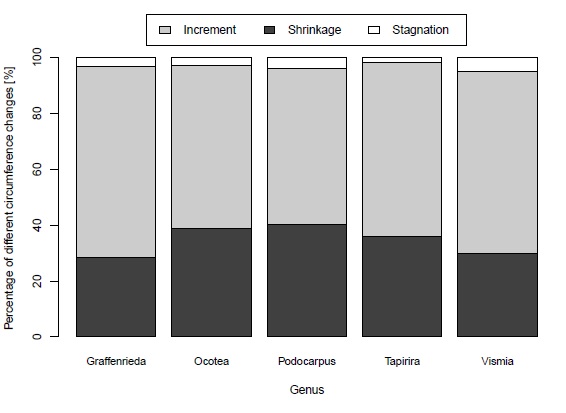

Supplement: FIGURE S1 — Percentage of different circumference changes (%) for different genera. [file Image_1.JPEG]

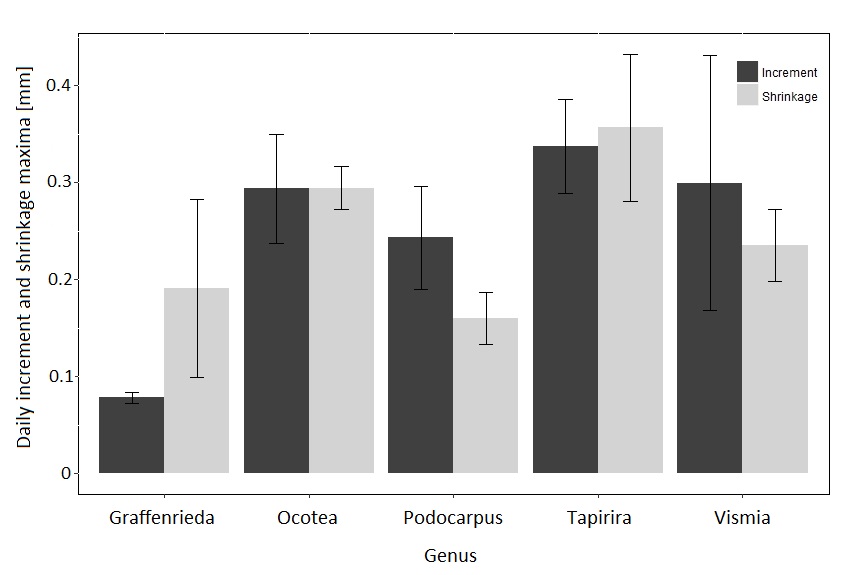

Supplement: FIGURE S2 — Daily increment and shrinkage maxima (mm) for different genera with standard deviations. [file Image_2.JPEG]

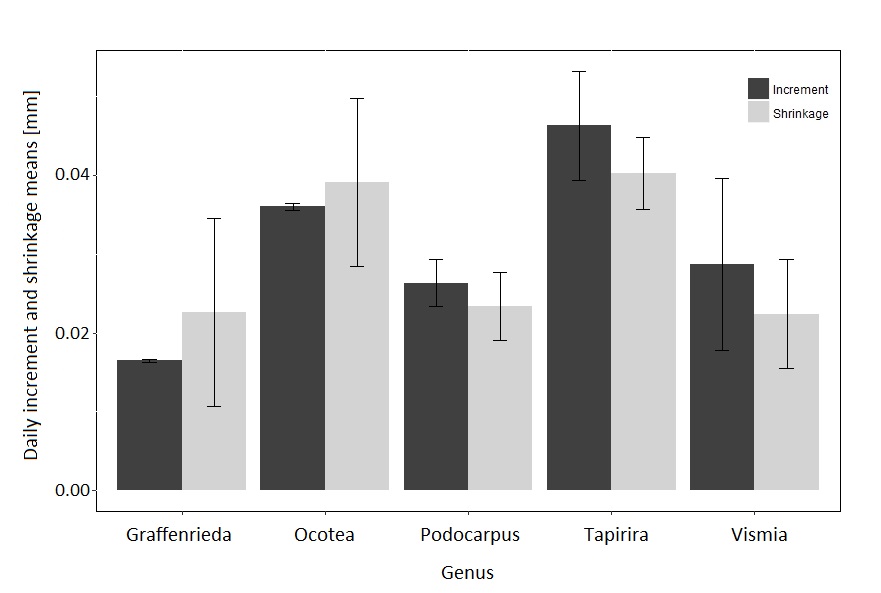

Supplement: FIGURE S3 — Daily increment and shrinkage means (mm) for different genera with standard deviations. [file Image_3.JPEG]

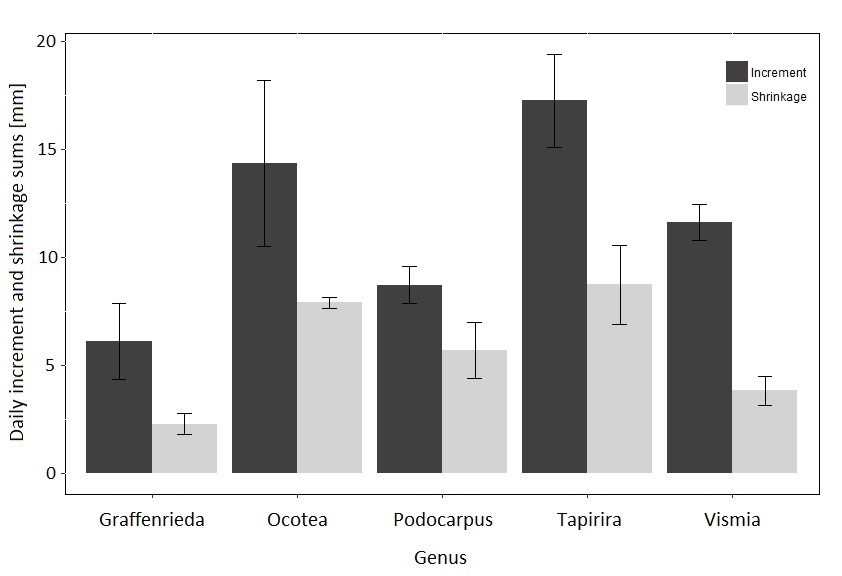

Supplement: FIGURE S4 — Daily increment and shrinkage sums (mm) for different genera with standard deviations. [file Image_4.JPEG]
